# Cytotoxic Activities and Molecular Mechanisms of the Beauvericin and Beauvericin G_1_ Microbial Products against Melanoma Cells

**DOI:** 10.3390/molecules25081974

**Published:** 2020-04-23

**Authors:** Haet Nim Lim, Jun-Pil Jang, Hee Jeong Shin, Jae-Hyuk Jang, Jong Seog Ahn, Hye Jin Jung

**Affiliations:** 1 Department of Life Science and Biochemical Engineering, Sun Moon University, Asan 31460, Korea; gotsla9210@naver.com (H.N.L.); gmlwjd903@naver.com (H.J.S.); 2 Department of Pharmaceutical Engineering and Biotechnology, Sun Moon University, Asan 31460, Korea; 3 Anticancer Agent Research Center, Korea Research Institute of Bioscience and Biotechnology, Cheongju 28116, Korea; jpjang@kribb.re.kr; 4 Natural Medicine Research Center, Korea Research Institute of Bioscience and Biotechnology, Cheongju 28116, Korea; 5 Department of Biomolecular Science, KRIBB School of Bioscience, Korea University of Science and Technology (UST), Daejeon 34141, Korea; 6 Genome-based BioIT Convergence Institute, Asan 31460, Korea

**Keywords:** melanoma, microbial product, beauvericin, beauvericin G_1_, apoptosis

## Abstract

Melanoma is the most serious type of skin cancer and remains highly drug-resistant. Therefore, the discovery of novel effective agents against melanoma is in high demand. Herein, we investigated the cytotoxic activities in melanoma cells and underlying molecular mechanisms of beauvericin (BEA) and its analogue beauvericin G_1_ (BEA G_1_), which are cyclohexadepsipeptides isolated from fungi. BEA and BEA G_1_ significantly suppressed the growth, clonogenicity, migration, and invasion of A375SM human melanoma cells and promoted caspase-dependent apoptosis through upregulation of death receptors, as well as modulation of pro- and anti-apoptotic Bcl-2 family members. Furthermore, the effects of BEA and BEA G_1_ were associated with the suppression of multiple molecular targets that play crucial roles in melanoma oncogenesis, including ERK, JNK, p38, NF-κB, STAT3, and MITF. Notably, the cytotoxic efficacy of BEA G_1_ against A375SM cells was stronger than that of BEA. These findings suggest that BEA and BEA G_1_ can be further investigated as potent cytotoxic natural compounds for the suppression of melanoma progression.

## 1. Introduction

The worldwide incidence of melanoma, a malignant skin cancer deriving from melanocytes, has been increasing more rapidly than other cancers. The prognosis for patients with malignant melanoma is bleak, with an average survival time of 6–9 months. As such, melanoma is responsible for 80% of skin cancer patient mortalities [1,2]. The development of melanoma is reported to be influenced by various genetic and epigenetic alterations. Mutations in the v-raf murine sarcoma viral oncogene homolog B1 (BRAF) gene, a serine threonine kinase, are identified in ~50% of malignant melanoma cases and lead to activation of the BRAF/mitogen-activated protein kinase (MEK)/extracellular signal-regulated kinase (ERK) signaling pathway, which regulates cell growth, proliferation, differentiation, and survival [3,4]. Consequently, chemotherapies using small molecule inhibitors of mutated BRAF, such as vemurafenib and dabrafenib, have generated positive clinical responses in approximately half of BRAF-mutated melanoma patients [5,6]. However, inherent or acquired resistance of melanoma cells to BRAF inhibitors necessitates the discovery of new and improved anticancer agents, which work by suppressing other relevant molecular targets that play critical roles in the development and progression of melanoma. 

Natural compounds with unique structures and properties represent attractive new drug leads. A recent report revealed that ~50% of all small molecule therapeutics are based on natural products or their derivatives [7]. Due to their special structures and valuable biological functions, cyclodepsipeptides have emerged as promising agents in the pharmaceutical field. Cyclic depsipeptides are a large family of peptide-related natural products, consisting of hydroxy and amino acids linked by amide and ester bonds. They show a wide range of biological properties, such as cytotoxic, phytotoxic, antimicrobial, antiviral, anthelmintic, insecticidal, antimalarial, immunosuppressant, and anti-inflammatory activities [8,9]. Several cyclodepsipeptides, including PF1022, aureobasidine, and enniatins, have been applied as potential pharmaceuticals and agrochemicals. The bioactive secondary metabolite beauvericin (BEA) was originally isolated from the entomopathogenic fungus *Beaveria bassiana* [10]. BEA, a cyclic hexadepsipeptide mycotoxin biosynthesized from N-methyl phenylalanine and 2-hydroxyisovaleric acid, is reported to exhibit diverse biological activities, including antimicrobial, insecticidal, antiviral, antiplatelet aggregation, ionophoric, anti-inflammatory, antimelanogenesis, and antitumor effects [11,12]. Mechanistic studies on the cytotoxic effects of BEA have shown that it induced apoptosis in several human cancer cells, such as those derived from the blood, lung, colon, liver, prostate, breast, pancreas, and brain. BEA promotes apoptosis through the intrinsic mitochondrial pathway, which involves the Bcl-2 family, cytochrome c release, and caspase-3 activation [13,14,15]. However, the cytotoxic effect of BEA against melanoma cells and its underlying molecular mechanism have not been reported. 

**Figure 1:**
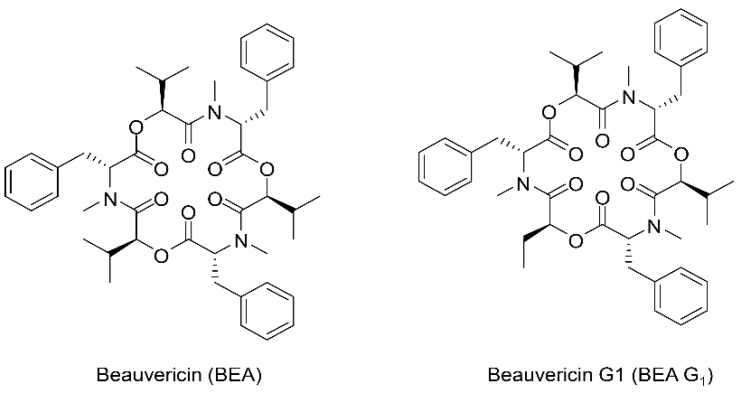
Chemical structures of BEA and BEA G_1_.

We recently isolated BEA and its known analogue BEA G_1_ from a fungus 16F003 (Figure 1). This study is the first report on the cytotoxic activities of BEA and BEA G_1_ and their involvement in apoptotic pathways in A375SM human melanoma cells.

## 2. Results

### 2.1. BEA and BEA G_1_ Inhibit the Growth of A375SM Melanoma Cells

**Figure 2:**
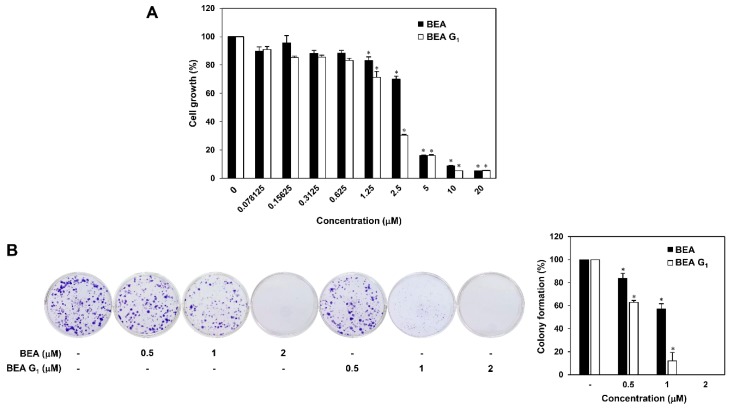
Growth inhibitory effects of BEA and BEA G_1_ on A375SM melanoma cells. (**A**) The effects of BEA and BEA G_1_ on the growth of A375SM cells. The cells were treated with increasing concentrations of BEA and BEA G_1_ (0–20 μM) for 72 h, and cell growth was measured by a 3-(4,5-dimethylthiazol-2-yl)-2,5-diphenyltetrazolium bromide (MTT) assay. (**B**) The effects of BEA and BEA G_1_ on the colony-forming ability of A375SM cells. The cells were treated with BEA and BEA G_1_ (0.5, 1, and 2 μM) and incubated for 10 days. The cell colonies were visualized by crystal violet staining and then counted. * = *p* < 0.05 versus the control.

To assess the effects of BEA and BEA G_1_ on the growth of melanoma cells, A375SM cells were treated with various concentrations (0–20 μM) of BEA and BEA G_1_ for 72 h, and the MTT assay was performed. As shown in Figure 2A, BEA and BEA G_1_ inhibited the growth of A375SM cells in a dose-dependent manner. Notably, the growth-inhibitory effect of BEA G_1_ (IC_50_ = 1.723 μM) was better than that of BEA (IC_50_ = 3.032 μM). 

We next examined the effects of BEA and BEA G_1_ on the colony-forming ability of A375SM cells. Clonogenic growth was dose-dependently suppressed by treatment with BEA or BEA G_1_ (Figure 2B). In addition, BEA G_1_ led to a more effective inhibition of colony formation in A375SM cells compared to BEA. These results indicate that BEA and BEA G_1_ possess potent antiproliferative activity against melanoma cells.

### 2.2. BEA and BEA G_1_ Inhibit the Migration of A375SM Melanoma Cells

**Figure 3:**
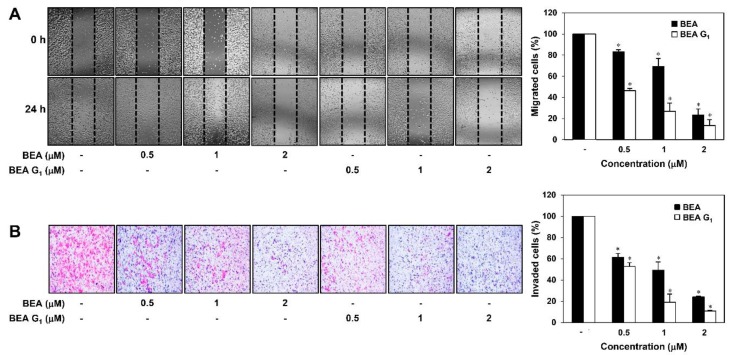
Migration inhibitory effects of BEA and BEA G_1_ on A375SM melanoma cells. (**A**) The effects of BEA and BEA G_1_ on the migration of A375SM cells. The migratory potential of A375SM cells was analyzed using a wound healing assay. The cells were treated with BEA and BEA G_1_ (0.5, 1, and 2 μM) for 24 h. Cells that migrated into the gap were counted using an optical microscope. Dotted black lines indicate the edge of the gap at 0 h. (**B**) The effects of BEA and BEA G_1_ on the invasion of A375SM cells. The invasiveness of A375SM cells was analyzed using Matrigel-coated polycarbonate filters. The cells were treated with BEA and BEA G_1_ (0.5, 1, and 2 μM) for 24 h. Cells that penetrated the filters were stained and counted using an optical microscope. * = *p* < 0.05 versus the control.

To evaluate whether BEA and BEA G_1_ affect the metastatic ability of melanoma cells, we first performed a wound healing assay. As shown in Figure 3A, treatment with BEA or BEA G_1_ for 24 h resulted in a dose-dependent decrease in the migration ability of A375SM cells in comparison with untreated control cells.

We further investigated the effects of BEA and BEA G_1_ on the invasive potential of A375SM cells using the Matrigel matrix-coated Transwell chamber system. As shown in Figure 3B, treatment with BEA and BEA G_1_ significantly reduced the invasiveness of A375SM cells. Moreover, suppression of both migration and invasion was stronger in response to BEA G_1_ treatment than to BEA treatment. However, the suppressive effects of BEA and BEA G_1_ on melanoma metastasis may be attributed in part to their inhibitory effects on melanoma cell growth. 

### 2.3. BEA and BEA G_1_ Induce Apoptosis in A375SM Melanoma Cells

**Figure 4:**
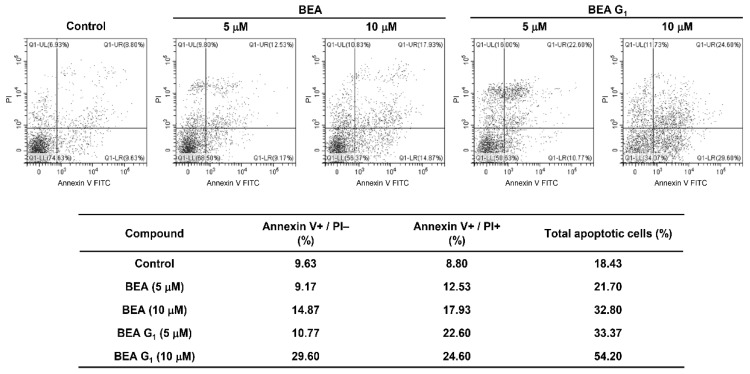
Effects of BEA and BEA G_1_ on the apoptotic cell death of A375SM melanoma cells. The cells were treated with BEA and BEA G_1_ (5 and 10 μM) for 24 h. Apoptotic cells were identified by flow cytometry following Annexin V-FITC and PI dual labeling.

To elucidate the cytotoxic mechanisms of BEA and BEA G_1_ in melanoma cells, cellular apoptosis was quantitatively measured using flow cytometric analysis following dual-labeling with Annexin V-FITC and propidium iodide (PI). Annexin V is a marker of early apoptosis, and PI is a marker of late apoptosis and necrosis. When A375SM cells were treated with BEA or BEA G_1_ for 24 h, the total number of early and late apoptotic cells was markedly increased in comparison to untreated control cells (Figure 4). 

**Figure 5:**
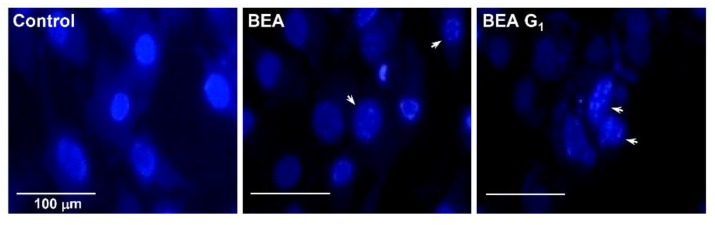
Effects of BEA and BEA G_1_ on the nuclear apoptosis of A375SM melanoma cells. The cells were treated with BEA and BEA G_1_ (5 μM) for 24 h. Changes in nuclear morphology were monitored by DAPI staining under a fluorescence microscope. Scale bar, 100 µm.

We next investigated whether BEA and BEA G_1_ cause nuclear apoptotic changes in A375SM melanoma cells. DAPI staining revealed that BEA and BEA G_1_ induced nuclear condensation in A375SM cells (Figure 5, arrows). 

**Figure 6:**
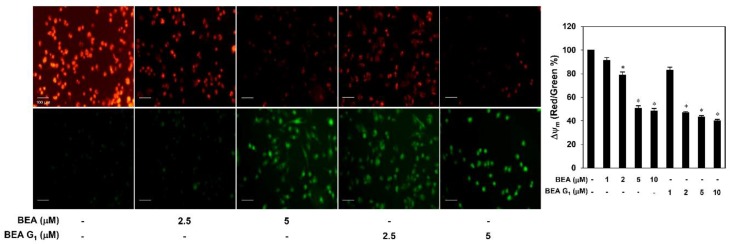
Effects of BEA and BEA G_1_ on MMP in A375SM melanoma cells. The cells were treated with BEA and BEA G_1_ (2.5 and 5 μM) for 24 h and stained with JC-1. The fluorescent images were obtained under a fluorescence microscope, and fluorescence intensity was detected using a multimode microplate reader. Scale bar, 100 µm. * = *p* < 0.05 versus the control.

Mitochondrial dysfunction is an early apoptotic event that includes a change in mitochondrial membrane potential (MMP) [16]. Thus, we measured the change in MMP following treatment of A375SM cells with BEA or BEA G_1_ using a fluorescent cationic probe (JC-1). As shown in Figure 6, the control cells exhibited a low level of green fluorescence and a high level of red fluorescence (a large negative MMP), whereas treatment with BEA or BEA G_1_ led to an increase in green fluorescence and a decrease in red fluorescence (a loss of MMP) in a dose-dependent manner. These data demonstrate that mitochondrial dysfunction may be closely related to apoptosis of A375SM cells induced by BEA and BEA G_1_. 

**Figure 7:**
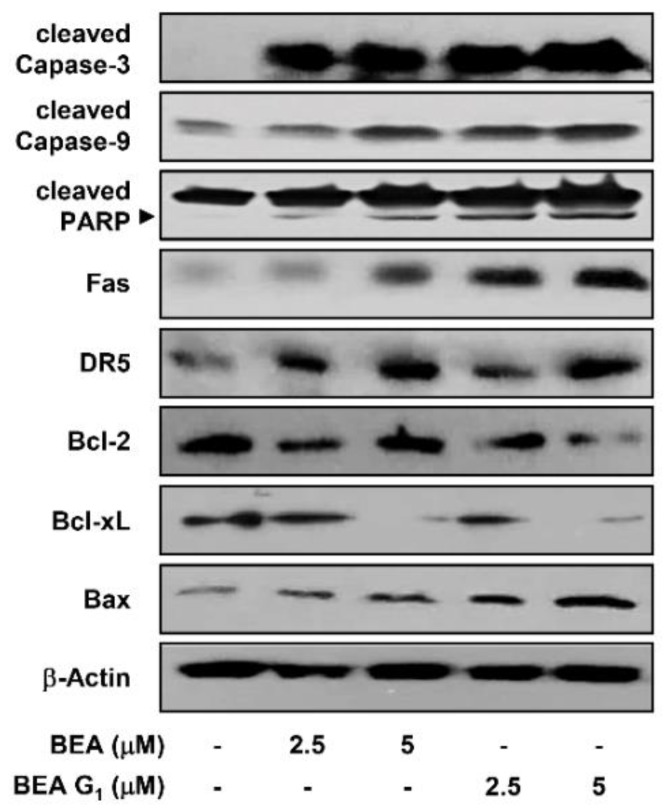
Effects of BEA and BEA G_1_ on the expression of apoptosis regulatory proteins in A375SM melanoma cells. The cells were treated with BEA and BEA G_1_ (2.5 and 5 μM) for 24 h, and protein levels were detected by Western blot analysis using specific antibodies. The levels of β-actin were used as an internal control. Arrowheads indicate cleaved forms.

To further characterize the apoptotic response induced by BEA and BEA G_1_, we examined whether they activate the intrinsic or extrinsic apoptotic pathways in A375SM cells. Western blot analysis revealed that BEA and BEA G_1_ increased the levels of activated caspase-3 and caspase-9 and subsequent cleavage of poly (ADP-ribose) polymerase (PARP), which is a substrate of activated caspase-3 (Figure 7). Furthermore, BEA and BEA G_1_ upregulated the expression of the Fas and DR5 death receptors. Bcl-2 family proteins control apoptosis by regulating outer mitochondrial membrane permeabilization [17]. Treatment with BEA or BEA G_1_ decreased the levels of antiapoptotic proteins including Bcl-2 and Bcl-xL and increased the expression of proapoptotic proteins such as Bax in A375SM cells. These results imply that BEA and BEA G_1_ induce apoptosis in melanoma cells through activation of both extrinsic and intrinsic apoptotic pathways. In particular, the apoptosis-inducing effect of BEA G_1_ was stronger than that of BEA. 

### 2.4. BEA and BEA G_1_ Downregulate Major Molecular Pathways Involved in Melanoma

**Figure 8:**
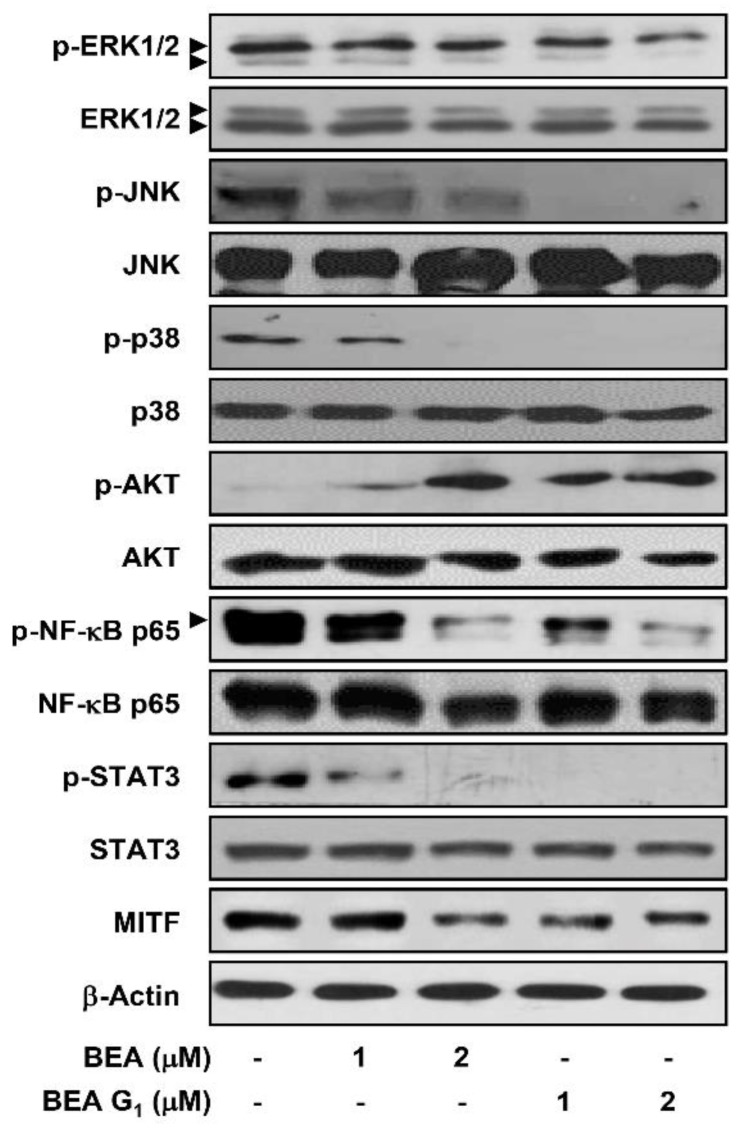
Effects of BEA and BEA G_1_ on the activation of multiple molecular targets involved in the pathogenesis of melanoma. A375SM cells were treated with BEA and BEA G_1_ (1 and 2 μM) for 24 h, and protein levels were detected by Western blot analysis using specific antibodies. The levels of β-actin were used as an internal control. Arrowheads indicate bands that correspond to the specific protein.

Although the BRAF/MEK/ERK pathway plays a critical role in melanoma progression, different molecular pathways, such as c-Jun N-terminal kinase (JNK), p38 MAPK, phosphatidylinositol 3-kinase (PI3K)/AKT, nuclear factor kappa B (NF-κB), signal transducer and activator of transcription 3 (STAT3), and microphthalmia-associated transcription factor (MITF) are also known to be constitutively active in malignant melanoma [18,19]. We, therefore, investigated whether BEA and BEA G_1_ affect multiple signaling pathways. As shown in Figure 8, BEA and BEA G_1_ effectively inhibited the phosphorylation of ERK1/2, JNK, p38, NF-κB p65, and STAT3, as well as MITF expression in A375SM cells. In contrast, BEA and BEA G_1_ increased levels of phosphorylated AKT. These results suggest that the cytotoxic activities of BEA and BEA G_1_ in A375SM cells are associated with downregulation of multiple molecular pathways. 

## 3. Discussion

Despite the diversity of therapeutic options available for melanoma, the high resistance of cancer cells to conventional treatments necessitates the search for new anticancer agents with greater efficacy [20,21]. Many natural cyclic peptides have shown potent cytotoxicity against cancer cell lines and excellent potential as cancer therapeutic agents [22]. In order to find effective natural compounds to treat melanoma, we investigated the cytotoxic effects in A375SM cells and the underlying molecular mechanisms of BEA and BEA G_1_, which are two fungal cyclic hexadepsipeptides. Previous studies have shown that BEA can induce apoptosis in several cancer cells, such as lung cancer, colon cancer, leukemia, and hepatoma cells [10,11,12,13,14]. However, there has been no report regarding the inhibitory activities of BEA and its analogue BEA G_1_ against malignant melanoma. BEA and BEA G_1_ potently inhibited the growth and migration of A375SM cells. The compounds induced caspase-dependent apoptosis in melanoma cells through upregulation of the Fas and DR5 death receptors and regulation of Bcl-2 family members (Bcl-2, Bcl-xL, and Bax). Our study also demonstrated that the effects of BEA and BEA G_1_ are associated with the suppression of molecular targets, which play crucial roles in melanoma oncogenesis, including ERK, JNK, p38, NF-κB, STAT3, and MITF. Notably, the cytotoxic effect of BEA G_1_ in melanoma cells was stronger than that of BEA.

Caspase-dependent apoptosis can be triggered by either the intrinsic mitochondrial pathway or the extrinsic death receptor pathway [23,24]. Mitochondria-mediated apoptosis is involved in a variety of cellular events, such as release of caspase activators, changes in electron transport, loss of mitochondrial membrane potential, and engagement of both proapoptotic and antiapoptotic Bcl-2 family proteins. Death-receptor-mediated apoptosis is triggered through the activation of death receptors of the tumor necrosis factor (TNF) family, including TNF receptor 1 (TNFR1), CD95 (APO-1, Fas), and TNF-related apoptosis-inducing ligand (TRAIL) receptors (DR4, DR5). Upon binding of death ligands to cognate receptors, the death-inducing signaling complex (DISC) is formed and activates the caspase cascade. In this study, we found that BEA and BEA G_1_ upregulated the expression of the Fas and DR5 death receptors and proapoptotic Bax, whereas they downregulated the expression of antiapoptotic Bcl-2 and Bcl-xL in A375SM cells. Subsequently, the caspase cascade, involving caspase-9, caspase-3, and PARP, was activated by these natural compounds. Therefore, BEA and BEA G_1_ seem to induce apoptosis in melanoma cells through activation of both the extrinsic and intrinsic apoptotic pathways. In addition, the apoptosis-inducing effect of BEA G_1_ was more potent than that of BEA.

Although the BRAF/MEK/ERK pathway is an obvious therapeutic target in BRAF-mutated melanoma, various other cellular pathways activated by oncogenic effectors, such as JNK, p38, PI3K/AKT, NF-κB, STAT3, and MITF, are also implicated in the development, progression, metastasis, and drug resistance of melanoma [18,19]. The JNK/c-Jun pathway is upregulated in a subset of melanoma cell lines, and co-treatment with RAF and JNK kinase inhibitors led to a synergistic induction of apoptosis [25]. The p38 MAPK pathway also increased the invasion of malignant melanoma cells through upregulation of matrix metallopeptidase (MMP-2) expression, indicating that inhibition of p38 MAPK may block signaling cascades related to melanoma metastasis [26]. Resistance to RAF inhibitors in BRAF^V600E^ melanoma frequently arises as a result of the upregulation of antiapoptotic pathways, such as PI3K/AKT [27]. Several studies have demonstrated that inhibition of the PI3K/AKT pathway sensitizes melanoma cells to BRAF/MEK/ERK pathway inhibitors [28]. In addition, components of the NF-κB family, such as p50 and p65, are overexpressed in melanoma cells, and upregulation of NF-κB is involved in the progression and metastasis of melanoma [29]. STAT3 is emerging as an important therapeutic target for melanoma, owing to its key role in promoting metastasis, angiogenesis, immune evasion, and stemness [30,31]. MITF is also known to regulate a number of genes involved not only in melanocyte differentiation and pigment formation, but also in the survival, growth, and metastasis of melanoma cells [32]. In metastatic melanoma, MITF amplification was associated with a decrease in patient survival, and disruption of MITF-sensitized melanoma to conventional chemotherapeutics [33]. Thus, simultaneous inhibition of multiple molecular pathways involved in the pathogenesis of malignant melanoma could help achieve maximal therapeutic efficacy. In our study, BEA and BEA G_1_ concurrently inhibited the activation of ERK1/2, JNK, p38 MAPK, NF-κB p65, STAT3, and MITF in A375SM melanoma cells, suggesting that the natural compounds may effectively inhibit aggressive melanoma by targeting multiple oncogenic molecular pathways. However, BEA and BEA G_1_ induced AKT activation. Therefore, a combination of the natural compounds with PI3K/AKT inhibitors may stimulate the cytotoxic activities of BEA and BEA G_1_ against melanoma cells.

A previous study has revealed that BEA G_1_ (IC_50_ = 5 μM) showed higher cytotoxicity than BEA (IC_50_ = 8 μM) in the metastatic prostate cancer cell line PC-3M [34]. Likewise, our results demonstrated that BEA G_1_ exhibits enhanced cytotoxic activity in comparison with BEA. Therefore, BEA G_1_ may possess superior pharmacological potential for treatment of malignant melanoma. Further investigations to identify the exact target of these natural compounds is required in order to fully understand the mechanisms behind their cytotoxic effects in melanoma cells, and structure–function studies would be informative as well.

## 4. Materials and Methods 

### 4.1. Materials

The Matrigel and Transwell chambers were obtained from BD Biosciences (San Jose, CA, USA) and Corning Costar (Acton, MA, USA), respectively. Antibodies against cleaved caspase-3, cleaved capase-9, PARP, Bax, Bcl-2, Bcl-xL, Fas, DR5, phospho-ERK1/2, ERK1/2, phospho-p38, p38, phospho-JNK, JNK, phospho-NF-κB, NF-κB, phospho-STAT3, STAT3, phospho-AKT, AKT, β-actin, rabbit IgG, and mouse IgG were purchased from Cell Signaling Technology (Danvers, MA, USA). The anti-MITF antibody was obtained from Abcam (Cambridge, MA, USA).

### 4.2. Culture Conditions for Fungus Strain

A fungus strain, 16F003, was isolated from a soil sample collected from Daejeon, Korea. The soil sample was suspended and diluted in distilled water. The diluted suspension was spread onto various agar plates and incubated at 28 °C until the fungal hypha appeared. 16F003 was grown on PD agar medium for 7 days and then inoculated into a 500 mL Erlenmeyer flask containing 75 mL of seed PD broth culture medium (24 g/L potato dextrose; BD Biosciences). Incubation was carried out at 28 °C for 3 days on a rotary shaker operating at 135 rpm. For large cultures, 1% of the pre-culture broth was inoculated into 40 × 1 L baffled Erlenmeyer flasks containing 250 mL of PD broth, which was cultured for 8 days at 28 °C on a rotary shaker with agitation at 135 rpm.

### 4.3. Isolation of BEA and BEA G_1_

The culture broth (10 L) was filtered and extracted three times with an equal volume of EtOAc, and the EtOAc layer was concentrated in vacuo. The EtOAc extract (2.6 g) was subjected to reverse-phase C_18_ vacuum column chromatography (RP-HPLC) with a stepwise solvent system of MeOH:H_2_O (20:80 to 100:0, each × 1 L). A fraction corresponding to 80% (80.4 mg) was further separated by RP-HPLC with CH_3_CN-H_2_O (70:30) to obtain BEA and BEA G_1_. The microbial products, BEA and BEA G_1_, were dissolved in dimethyl sulfoxide (DMSO) at a concentration of 100 mM as a stock solution. In all cell-based assays performed in this study, the stock solution was further diluted with culture medium for appropriate working doses. The negative control groups were treated with equal volumes of DMSO. 

### 4.4. Human Cell Culture 

The A375SM human melanoma cell line was obtained from the Korean Cell Line Bank (KCLB No. 80004). The identity of the A375SM cell line was confirmed by STR profiling (D3S1358: 15,17; vWA: 16,17; FGA: 20,23; Amelogenin: X; TH01: 8; TPOX: 8,10; CSF1PO: 11,12; D5S818: 12; D13S317: 11; D7S820: 9). The cells were grown in Dulbecco’s modified Eagle medium (DMEM; Gibco, Grand Island, NY, USA), supplemented with 10% fetal bovine serum (FBS; Gibco) and 1% penicillin–streptomycin–amphotericin B (Lonza, Walkersville, MD, USA), and then maintained at 37 °C in a humidified 5% CO_2_ incubator. 

### 4.5. Cell Growth Assay 

Cell growth was examined using the 3-(4,5-dimethylthiazol-2-yl)-2,5-diphenyltetrazolium bromide (MTT) colorimetric assay. A375SM cells were seeded in a 96-well culture plate at a density of 2 × 10^3^ cells/well. After 24 h of incubation, various concentrations of BEA and BEA G_1_ were added to each well. After 72 h of incubation, 50 μL of MTT solution (2 mg/mL; Sigma-Aldrich, Saint Louis, MO, USA) was added to each well, and the cells were incubated for 3 h at 37 °C. To dissolve formazan crystals, the culture medium was removed and an equal volume of DMSO was added to each well. The absorbance of each well was determined at a wavelength of 540 nm using a microplate reader (Thermo Fisher Scientific, Vantaa, Finland). The IC_50_ values from obtained data were analyzed using the curve-fitting program GraphPad Prism 5 (GraphPad Software, La Jolla, CA, USA).

### 4.6. Colony Formation Assay 

A375SM cells were seeded in a 6-well cell culture plate at a density of 1 × 10^3^ cells/well. After 24 h of incubation, the cells were treated with BEA and BEA G_1_ for 10 days. The formed colonies were fixed with 4% formaldehyde and stained with 0.5% crystal violet solution. The number of colonies in each well was counted and the percentage of compound-treated colonies relative to DMSO-treated control colonies was calculated.

### 4.7. Chemoinvasion Assay 

Cell invasion was examined using Transwell chamber inserts with a pore size of 8.0 μm. The lower side of the polycarbonate filter was coated with 10 μL of gelatin (1 mg/mL), and the upper side was coated with 10 μL of Matrigel (3 mg/mL). A375SM cells (1 × 10^5^) were seeded in the upper chamber of the filter, and BEA and BEA G_1_ were added to the lower chamber filled with the medium. After 24 h of incubation, the cells were fixed with methanol and stained with hematoxylin and eosin. The total number of cells that invaded the lower chamber of the filter was counted using an optical microscope (Olympus, Center Valley, PA, USA). 

### 4.8. Cell Migration Assay 

A375SM cells were seeded in a 24-well cell culture plate at a density of 2 × 10^5^ cells/well and grown to 90% confluence. The confluent monolayer cells were scratched using a pipette tip and each well was washed with phosphate-buffered saline (PBS) to remove non-adherent cells. The cells were treated with BEA and BEA G_1_ and then incubated for 24 h. The perimeter of the central cell-free zone was determined using an optical microscope (Olympus).

### 4.9. Apoptosis Analysis 

A375SM cells (3 × 10^5^ cells/dish) were treated with BEA and BEA G_1_ for 24 h. The cells were harvested, washed with PBS, and stained with Annexin V-FITC and PI according to the manufacturer’s instructions for the ApopNexin Annexin V FITC Apoptosis kit (Merck Millipore, Darmstadt, Germany). The stained cells were analyzed by flow cytometry (BD Biosciences). 

### 4.10. DAPI Fluorescent Staining 

A375SM cells were seeded in a 24-well black cell culture plate at a density of 5 × 10^4^ cells/well. After treatment with BEA and BEA G_1_ for 24 h, the cells were washed with PBS and then stained with 4′,6-diamidino-2-phenylindole (DAPI, 5 μg/mL; Sigma-Aldrich) for 10 min at room temperature. Nuclear morphology was observed using a fluorescence microscope (Optinity KI-2000F; Korea Lab Tech, Seongnam, Korea).

### 4.11. MMP Determination 

MMP was detected using the fluorescent lipophilic dye JC-1 (5,5′,6,6′-tetra-chloro-1,1′,3,3′-tetraethylbenzimidazol-carbocyanine iodide; Sigma-Aldrich). At hyperpolarized membrane potentials, this dye forms a red fluorescent J-aggregate, whereas at depolarized membrane potentials, this dye remains in its green fluorescent monomeric form. A375SM cells were seeded in a 24-well black cell culture plate at a density of 5 × 10^4^ cells/well. After treatment with BEA and BEA G_1_ for 24 h, the cells were incubated with 10 μg/mL of JC-1 for 20 min and washed with PBS. The images were obtained using an Optinity KI-2000F fluorescence microscope and the fluorescence intensity was detected using a multimode microplate reader (Biotek, Inc., Winooski, VT, USA).

### 4.12. Western Blot Analysis 

Cell lysates were separated by 10% sodium dodecyl sulfate-polyacrylamide gel electrophoresis (SDS-PAGE), and the separated proteins were transferred to polyvinylidene difluoride (PVDF) membranes (Millipore, Billerica, MA, USA) using standard electroblotting procedures. The blots were blocked and immunolabeled with primary antibodies against cleaved caspase-3, cleaved capase-9, PARP, Bax, Bcl-2, Bcl-xL, Fas, DR5, phospho-ERK1/2, ERK1/2, phospho-p38, p38, phospho-JNK, JNK, phospho-NF-κB, NF-κB, phospho-STAT3, STAT3, phospho-AKT, AKT, MITF, and β-actin overnight at 4 °C. Immunolabeling was detected with an enhanced chemiluminescence (ECL) kit (Bio-Rad Laboratories, Hercules, CA, USA), according to the manufacturer’s instructions.

### 4.13. Statistical Analysis 

The data are presented as the mean ± standard deviation (SD) of three independent experiments. Student’s *t*-tests were used to determine statistical significance between the control and the test groups. A *p*-value of <0.05 was considered to indicate a statistically significant difference.

## 5. Conclusions

This is the first study to identify cytotoxic effects and the underlying molecular mechanisms of BEA and BEA G_1_ with regards to malignant melanoma. The natural microbial products potently suppressed the growth and migration of A375SM melanoma cells. BEA and BEA G_1_ induced caspase-dependent apoptosis through activation of both the extrinsic and intrinsic apoptotic pathways. Furthermore, the cytotoxic activities of BEA and BEA G_1_ in A375SM cells were associated with downregulation of multiple molecular pathways, including the ERK, JNK, p38, NF-κB, STAT3, and MITF pathways. Notably, BEA G_1_ exhibited greater cytotoxic effects than BEA. In conclusion, our findings suggest that BEA and BEA G_1_ may be further investigated as potent natural compounds to treat malignant melanoma.

## References

[1] Miller K.D., Nogueira L., Mariotto A.B., Rowland J.H., Yabroff K.R., Alfano C.M., Jemal A., Kramer J.L., Siegel R.L. (2019). Cancer treatment and survivorship statistics, 2019. CA A Cancer J. Clin..

[2] Marghoob A.A., Kopf A.W., Rigel D.S., Bart R.S., Friedman R.J., Yadav S., Abadir M., Sanfilippo L., Silverman M.K., Vossaert K.A. (1994). Risk of Cutaneous Malignant Melanoma in Patients with ’Classic’ Atypical-Mole Syndrome. Arch. Dermatol..

[3] Tsao H., Chin L., Garraway L.A., Fisher D.E. (2012). Melanoma: from mutations to medicine. Genes Dev..

[4] Ascierto P.A., Kirkwood J.M., Grob J.-J., Simeone E., Grimaldi A.M., Maio M., Palmieri G., Testori A., Marincola F.M., Mozzillo N. (2012). The role of BRAF V600 mutation in melanoma. J. Transl. Med..

[5] Niezgoda A., Niezgoda P., Czajkowski R. (2015). Novel Approaches to Treatment of Advanced Melanoma: A Review on Targeted Therapy and Immunotherapy. BioMed Res. Int..

[6] Shtivelman E., Davies M.A., Hwu P., Yang J., Lotem M., Oren M., Flaherty K.T., Fisher D.E. (2014). Pathways and therapeutic targets in melanoma. Oncotarget.

[7] Chinembiri T.N., Du Plessis L., Gerber M., Hamman J.H., Du Plessis J. (2014). Review of Natural Compounds for Potential Skin Cancer Treatment. Molecules.

[8] Wang X., Gong X., Li P., Lai D., Zhou L. (2018). Structural Diversity and Biological Activities of Cyclic Depsipeptides from Fungi. Molecules.

[9] Sivanathan S., Scherkenbeck J. (2014). Cyclodepsipeptides: A Rich Source of Biologically Active Compounds for Drug Research. Molecules.

[10] Wang Q., Xu L. (2012). Beauvericin, a Bioactive Compound Produced by Fungi: A Short Review. Molecules.

[11] Wu Q., Patocka J., Nepovimova E., Ramalho T.C. (2018). A Review on the Synthesis and Bioactivity Aspects of Beauvericin, a Fusarium Mycotoxin. Front. Pharmacol..

[12] Lee S.E., Park S.-H., Oh S.W., Yoo J.A., Kwon K., Park S.J., Kim J., Lee H.S., Cho J., Lee J. (2018). Beauvericin inhibits melanogenesis by regulating cAMP/PKA/CREB and LXR-α/p38 MAPK–mediated pathways. Sci. Rep..

[13] Jow G.-M., Chou C.-J., Chen B.-F., Tsai J.-H. (2004). Beauvericin induces cytotoxic effects in human acute lymphoblastic leukemia cells through cytochrome c release, caspase 3 activation: the causative role of calcium. Cancer Lett..

[14] Lin H.-I., Lee Y.-J., Chen B.-F., Tsai M.-C., Lu J.-L., Chou C.-J., Jow G.-M. (2005). Involvement of Bcl-2 family, cytochrome and caspase 3 in induction of apoptosis by beauvericin in human non-small cell lung cancer cells. Cancer Lett..

[15] Mallebrera B., Prosperini A., Font G., Ruiz M.-J. (2017). In vitro mechanisms of Beauvericin toxicity: A review. Food Chem. Toxicol..

[16] Ly J.D., Grubb D.R., Lawen A. (2003). The mitochondrial membrane potential (deltapsi(m)) in apoptosis; an update. Apoptosis.

[17] Martinou J.-C., Youle R.J. (2011). Mitochondria in Apoptosis: Bcl-2 Family Members and Mitochondrial Dynamics. Dev. Cell.

[18] Palmieri G., Ombra M., Colombino M., Casula M., Sini M., Manca A., Paliogiannis P., Ascierto P.A., Cossu A.G.M. (2015). Multiple Molecular Pathways in Melanomagenesis: Characterization of Therapeutic Targets. Front. Oncol..

[19] Smalley K.S., Haass N.K., Brafford P., Lioni M., Flaherty K.T., Herlyn M. (2006). Multiple signaling pathways must be targeted to overcome drug resistance in cell lines derived from melanoma metastases. Mol. Cancer Ther..

[20] Kalal B., Upadhya D., Pai V.R. (2017). Chemotherapy Resistance Mechanisms in Advanced Skin Cancer. Oncol. Rev..

[21] Kozar I., Margue C., Rothengatter S., Haan C., Kreis S. (2019). Many ways to resistance: How melanoma cells evade targeted therapies. Biochim. Biophys. Acta (BBA) Bioenerg..

[22] Abdalla M.A., McGaw L.J. (2018). Natural Cyclic Peptides as an Attractive Modality for Therapeutics: A Mini Review. Molecules.

[23] Jan R., Chaudhry G.-E.-S. (2019). Understanding Apoptosis and Apoptotic Pathways Targeted Cancer Therapeutics. Adv. Pharm. Bull..

[24] Baig S., Seevasant I., Mohamad J., Mukheem A., Huri H.Z., Kamarul T. (2016). Potential of apoptotic pathway-targeted cancer therapeutic research: Where do we stand?. Cell Death Dis..

[25] Fallahi-Sichani M., Moerke N.J., Niepel M., Zhang T., Gray N.S., Sorger P.K. (2015). Systematic analysis of BRAF V 600E melanomas reveals a role for JNK/c-Jun pathway in adaptive resistance to drug-induced apoptosis. Mol. Syst. Boil..

[26] Denkert C., Siegert A., LeClere A., Turzynski A., Hauptmann S. (2002). An inhibitor of stress-activated MAP-kinases reduces invasion and MMP-2 expression of malignant melanoma cells. Clin. Exp. Metastasis.

[27] Davies M.A. (2012). The Role of the PI3K-AKT Pathway in Melanoma. Cancer J..

[28] Caporali S., Alvino E., Lacal P.M., Levati L., Giurato G., Memoli D., Caprini E., Cappellini G.C.A., D’Atri S. (2016). Targeting the PI3K/AKT/mTOR pathway overcomes the stimulating effect of dabrafenib on the invasive behavior of melanoma cells with acquired resistance to the BRAF inhibitor. Int. J. Oncol..

[29] Madonna G., Ullman C.D., Gentilcore G., Palmieri G., Ascierto P.A. (2012). NF-κB as potential target in the treatment of melanoma. J. Transl. Med..

[30] Lesinski G.B. (2013). The potential for targeting the STAT3 pathway as a novel therapy for melanoma. Futur. Oncol..

[31] Wu K.J., Huang J.M., Zhong H.J., Dong Z.Z., Vellaisamy K., Lu J.J., Chen X.P., Chiu P., Kwong D.W.J., Han Q.B. (2017). A natural product-like JAK2/STAT3 inhibitor induces apoptosis of malignant melanoma cells. PLoS ONE.

[32] Levy C., Khaled M., Fisher D.E. (2006). MITF: master regulator of melanocyte development and melanoma oncogene. Trends Mol. Med..

[33] Aida S., Sonobe Y., Tanimura H., Oikawa N., Yuhki M., Sakamoto H., Mizuno T. (2017). MITF suppression improves the sensitivity of melanoma cells to a BRAF inhibitor. Cancer Lett..

[34] Xu Y., Zhan J., Wijeratne E.M.K., Burns A.M., Gunatilaka A.A.L., Molnar I. (2007). Cytotoxic and Antihaptotactic Beauvericin Analogues from Precursor-Directed Biosynthesis with the Insect PathogenBeauveria bassianaATCC 7159. J. Nat. Prod..

